# Supercritical Fluid Extraction of Ergosterol from *Lentinula edodes* and *Pleurotus ostreatus*: Optimization and Synergistic Effects of Biomass Pre-Treatments

**DOI:** 10.3390/molecules31122067

**Published:** 2026-06-12

**Authors:** Rita Faustino, António Ferreira, Maria Rosário Bronze, Naiara Fernández

**Affiliations:** 1 Instituto de Biologia Experimental e Tecnológica, Apartado 12, 2881-901 Oeiras, Portugal; rita.faustino@ibet.pt (R.F.); antoniof@ibet.pt (A.F.); mbronze@ibet.pt (M.R.B.); 2 Instituto de Tecnologia Química e Biológica António Xavier, Universidade Nova de Lisboa, Av. República, 2780-157 Oeiras, Portugal; 3 FFULisboa, Faculdade de Farmácia da Universidade de Lisboa, Av. Professor Gama Pinto, 1649-003 Lisboa, Portugal

**Keywords:** ergosterol, mushroom, supercritical fluid extraction, optimization, ultrasound pre-treatment, microwave pre-treatment, enzymatic pre-treatment

## Abstract

Ergosterol (ERG) is a bioactive sterol found in fungal cell membranes with reported cholesterol-lowering, antioxidant, and antitumor properties. Supercritical Fluid Extraction (SFE) conditions were optimized for *Lentinula edodes* (shiitake) using Response Surface Methodology and subsequently applied to *Pleurotus ostreatus* (oyster). Optimized SFE (690 bar, 69.8 °C, no co-solvent) produced significantly more concentrated ERG extracts than Soxhlet extraction for both species—280.57 ± 10.80 mg_ERG_/g_extract_ for shiitake and 95.87 ± 7.18 mg_ERG_/g_extract_ for oyster—corresponding to a 107% and 65% increase, respectively. Three biomass pre-treatments—ultrasound, microwave, and enzymatic—were evaluated in combination with SFE. Enzymatic pre-treatment with chitinase significantly improved ERG concentration: 337.53 ± 23.12 mg_ERG_/g_extract_ for shiitake. These results obtained after analysis of samples by GC-MS demonstrate that high-pressure SFE combined with chitinase pre-treatment is an effective strategy for producing ERG-rich extracts from fungal matrices.

## 1. Introduction

Cardiovascular diseases are the leading cause of death worldwide, strongly associated with risk factors such as hypercholesterolemia, smoking, inadequate diets and sedentary lifestyles [1].

Numerous studies have demonstrated the bioactive properties of ergosterol (ERG), a predominant sterol found in fungal plasma membranes inside the cell wall [2], including cholesterol-lowering, antioxidant and antitumor activities [3,4,5,6].

Mushrooms are among the fungal species richest in ERG. Although roughly two thousand edible species exist, only a few are cultivated and industrially processed, including *Lentinula edodes* (shiitake) and *Pleurotus ostreatus* (oyster) [7,8]. Mushroom cultivation generates significant amounts of waste, mainly spent mushroom substrate, stalks and misshapen mushrooms [9,10], translating to over 3 billion kilograms of waste annually across the EU [11]. These by-products may still contain substantial amounts of ERG that remain unutilized [12], and its recovery prevents the loss of a valuable compound, while contributing to circular economy principles, promoting more sustainable and resource-efficient mushroom production systems.

Being a lipophilic compound, ERG extraction depends on solvent non-polarity, capacity to penetrate the chitin-based fungal cell walls, temperature and mass transfer [13]. Soxhlet (SOX) extraction is a well-established conventional technique used for the ERG extraction from mushrooms [14,15,16], but presents drawbacks, including high organic solvent consumption, high temperatures and long extraction times. Consequently, greener emerging extraction technologies have been investigated as alternative methods.

Supercritical Fluid Extraction (SFE) has been studied extensively in the last decade, due to its operational advantages over conventional extraction methods. Using solvents in a supercritical state, combining liquid-like density with gas-like viscosity and intermediate diffusivity, SFE enables efficient extraction of intracellular compounds [17,18]. Furthermore, by adjusting pressure and temperature, the selectivity of the process can be finely tuned toward the target compound. Carbon dioxide (CO_2_) is the most used supercritical fluid, owing to its low toxicity, environmental safety, affordability, and moderate critical temperature (31.2 °C) [19,20].

SFE has been investigated for ERG extraction from mushrooms using different operating conditions. Gil-Ramírez et al. [19] evaluated pressures of 90, 180 and 300 bar with ethanol (EtOH) as a co-solvent for sterol-enriched fractions. In a subsequent study, the same author [3] successfully produced ERG-rich extracts using SFE at 300 bar, 40 °C for 3 h. Morales et al. [21] reported high ERG percentage in the extracts at operating conditions of 350 bar, 70 °C for 3 h, and, in another study [22], applied a sequential extraction approach, reporting that the SFE fraction (350 bar, 40 °C for 3 h) yielded the greatest ERG recovery. Morales et al. [23] also tested the influence of extraction time on the bioactive compound’s extraction from mushrooms, including ERG, performing SFE at 225 bar and 40 °C. Almeida et al. [24] achieved a maximum ERG purity operating at 100 bar and 50 °C. Despite this substantial amount of work, studies have employed a relatively narrow pressure range. Research exploring very high pressures, which substantially increases CO_2_ density and therefore has potential to markedly enhance extraction efficiency, remains limited.

Beyond optimizing extraction techniques, effective disruption of the rigid fungal cell wall is equally important to potentiate the release of intracellular compounds [25]. Optimized extraction and biomass pre-treatments act synergistically, delivering greater efficiency and compound enrichment than either strategy alone. Cell wall disruption methods investigated include ultrasound (US), microwaves (MWs) and enzymes (EZs) [25,26,27].

US pre-treatment offers an easy, inexpensive and scalable approach, widely used in mushrooms for drying improvement [28], water distribution and microstructure studies [29]. To our knowledge, no published data demonstrates its synergy with other extraction techniques for ERG recovery. MW pre-treatment also offers a rapid, automated, and cost-efficient approach, successfully applied in other biomasses [25,30,31], yet, to the best of our knowledge, no studies have reported its use as a pre-treatment in mushrooms for enhancing ERG extraction. EZ is a promising pre-treatment due to its simple equipment requirements [25] and aqueous process conditions, and is widely used to enhance bioactive compounds extraction [25,32,33]. However, its application to mushrooms [34], for improving ERG extraction, remains limited.

Liu et al. [35] proposed the use of steam explosion as a pre-treatment to improve ERG accessibility in *Flammulina velutipes*, followed by ultrasound-assisted saponification extraction. The results demonstrated that the combination of cell wall disruption prior to extraction significantly improved ERG extraction yield and diffusion kinetics, further supporting the rationale that pre-treatment of fungal biomass can act synergistically with the subsequent extraction step to enhance ERG recovery. The present work aimed to obtain ERG-rich extracts from shiitake and oyster mushrooms by combining biomass pre-treatments with SFE. The SFE process was optimized through Response Surface Methodology to identify the conditions that maximize ERG concentration in the obtained extracts. The synergistic effects between SFE and the US, MW and EZ pre-treatments, to potentially improve extract ERG-richness, were evaluated.

## 2. Results and Discussion

### 2.1. Conventional Extraction—Soxhlet

Two types of mushrooms were submitted to a SOX extraction with *n*-hexane for their characterization. In this section, *Lentinula edodes* is hereafter designated as shiitake and *Pleurotus ostreatus* as oyster, for simplicity and brevity. Table 1 demonstrates the results obtained for the mass extraction yield, concentration of ERG in the extract and the overall ERG yield.

The mass extraction yields of both mushrooms were similar. Shiitake produced extracts with higher ERG concentration, which may be attributed to its intrinsically higher ERG content relative to oyster mushroom [36,37]. Despite these differences, the overall ERG yield was comparable between the two species, with some slight differences.

The shiitake and oyster mushrooms yielded higher ERG values than those reported by Barreira et al. [38], 217 ± 2 mg_ERG_/100 g_DM_ for shiitake and 104 ± 1 mg_ERG_/100 g_DM_ for oyster, extracted with SOX with the same solvent. Wang et al. [39] reported lower values when using shiitake and SOX with the same solvent, 185.21 ± 10.64 mg_ERG_/100 g_DM_. In addition, the ERG yield obtained for oyster mushroom in this study is comparable to the value reported by Silva et al. [40], 290.90 mg_ERG_/100 g_DM_, when using a different extraction technique, and Taofiq et al. [41] reported a less concentrated ERG extract from oyster mushroom using SOX with EtOH, 78.20 ± 0.54 mg_ERG_/g_extract_.

The differences between the literature and our work and the variability of ERG yield may be attributed to the fact that ERG content is dependent on several factors, including cultivation practices, which can significantly enhance or reduce its concentration, and exposure to UV light can convert ERG into ergocalciferol (vitamin D_2_) [42]. Particle size may also have influenced ERG extraction, as the particles used in this study (63–250 µm) were smaller than those reported in the literature (420–850 µm), enhancing the surface area, and the range was optimized based on preliminary SOX extraction results. These factors likely contributed to the effective preservation and higher recovery of ERG during SOX extraction.

### 2.2. Supercritical Fluid Extraction with CO_2_

SFE was optimized using the shiitake mushroom as a model, due to its larger quantity available for our research compared with the oyster mushroom, and its frequent use in similar extraction studies reported in the literature. The optimized SFE process was compared with SOX extraction to assess whether it produces extracts with higher ERG concentrations.

#### 2.2.1. SFE Optimization

To optimize the extraction procedure, a Design of Experiments (Table 2) was planned, and the experiments were performed in a random order, defined by the software. For modeling and optimizing the responses, a Response Surface Methodology (RSM) was performed. The process variables were temperature (40–70 °C), pressure (350–800 bar) and the use of EtOH (0–10 mL/min) as co-solvent. The responses that were evaluated were mass extraction yield (g_extract_/100 g_DM_) and ERG concentration in the extracts (mg_ERG_/g_extract_) and are presented in Table 2.

**Figure 1:**
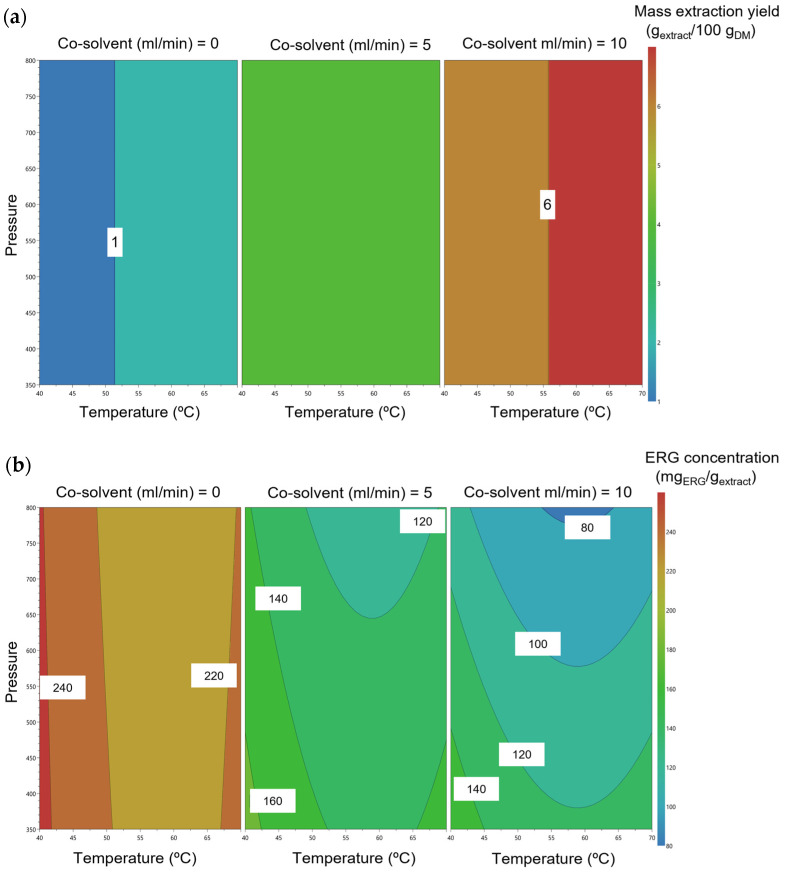
Response surface contour plot fitted to the mass extraction yield (upper panels, (**a**)) and to ERG concentration in the extract (lower panels, (**b**)) as a function of pressure (350–800 bar), temperature (40–70 °C) and co-solvent flow (EtOH 0–10 mL/min). Numerical contour labels (white boxes) indicate predicted response values. The numbered annotations above the panels correspond to the co-solvent levels used in the visualization (0, 5, and 10 mL/min).

After the experiments, RSM was applied. The significance of model terms (pressure, temperature, co-solvent, quadratic terms, and interactions) was included to provide a clearer interpretation of factor contributions and model adequacy (Table S1, Supplementary Information). The predictive performance and robustness of the developed models were evaluated through model fitting and validation parameters, including R^2^, adjusted R^2^, Q^2^, model validity reproducibility and ANOVA analysis for each response (Table S2, Supplementary Information). Overall, the models showed satisfactory goodness-of-fit and predictive ability, supporting their suitability for response optimization within the experimental domain studied. Figure 1 shows the response contour plots for each response.

For mass extraction yield (Figure 1a), co-solvent was the dominant factor, with temperature showing a borderline contribution, while pressure had a negligible effect. The highest mass extraction yield (6.48 g_extract_/100 g_DM_) was obtained under the conditions of Experiment 11 (575 bar, 55 °C, EtOH 10 mL/min), which combines a high supercritical CO_2_ density with high co-solvent quantity, enhancing both solvating power and diffusion. When no co-solvent was used, temperature had a slight positive impact on mass extraction yield, with higher temperatures favoring extractability through enhanced molecular diffusion [19,21,43]. The lowest yield (0.78 g_extract_/100 g_DM_) occurred in Experiment 13 (800 bar, 40 °C, EtOH 0 mL/min). At these conditions, supercritical CO_2_ density is very high, and its non-polar nature limits its ability to solubilize polar compounds, resulting in a lower mass yield.

The contour plot for ERG concentration (Figure 1b) shows a clear decrease in the target compound content as co-solvent flow increases. This factor was the only statistically significant factor for this response, as it was also observed for mass extraction yield. When no co-solvent is added, the response surface suggested a slight trend to higher pressures combined with lower temperatures to favor ERG concentration, although neither effect reached statistical significance (Table S1). The highest ERG content occurred in Experiment 13 (800 bar, 40 °C, EtOH 0 mL/min), 243.57 mg_ERG_/g_extract_. At these conditions, the very high-density SC-CO_2_ enhanced the extraction and solubility of ERG, while maintaining selectivity by limiting the extraction of polar compounds, as observed in the contour plots. These results are in accordance with what is reported in the literature [19,21]. Conversely, the lowest ERG concentration, 83.92 mg_ERG_/g_extract_, was observed in Experiment 14 (800 bar, 40 °C, EtOH 10 mL/min), where the addition of EtOH reduced the extract’s selectivity by promoting the co-extraction of other compounds alongside ERG.

Overall, co-solvent addition was the dominant factor governing mass extraction yield and ERG concentration in the extracts. The *p*-values associated with each factor and their impact for each response are presented in the Supplementary Information (Table S1).

Based on the analysis of the DoE results, an inverse trend was observed between mass extraction yield and ERG concentration in the extracts, and the maximization of each response was not deemed feasible. Since the main objective of this work is to produce highly concentrated ERG extracts, it is expected that the mass yield would be low. However, a balance must be achieved: the extract should remain sufficiently enriched in ERG while still providing enough total mass to allow practical handling and downstream processing.

To identify the optimal conditions for maximizing ERG concentration in shiitake mushroom extracts, the “Optimizer” tool within the MODDE software was employed. ERG concentration was selected as the response to be maximized, and the mass extraction yield was left as “predicted”, but with the constraint that it should not be lower than 1 g_extract_/100 g_DM_ to ensure sufficient extract quantity for practical handling.

The optimal conditions suggested by the model were 69.8 °C, 690 bar, and 0.023 mL/min of co-solvent. The higher temperature reflects its borderline positive contributions to mass extraction yield, helping to maintain extractability above the defined minimum, at very low co-solvent flow. Although pressure was not statistically significant, 690 bar was retained among the optimal conditions based on the optimization output, as this result is consistent with the mechanistic advantage of supercritical CO_2_ density for extract selectivity at elevated pressure, and with the slight trend observed in the response contour plot.

These optimized conditions were then experimentally tested, and the observed results were compared with the model’s predictions. As the co-solvent pump cannot produce that low flow, it was decided that no co-solvent would be added, further enhancing the ERG concentration. Table 3 summarizes the outcomes.

The observed mass extraction yield was lower than the predicted value, probably due to the fact that no co-solvent was added, but remained within the model 95% prediction interval. This indicates that the deviation is acceptable and the model reliably describes the extraction behavior for mass yield, showing limited sensitivity to small variation in operating conditions. However, the combination of high temperatures and high pressure might have contributed to maintaining mass extract yield above the defined minimum, consistent with previously reported effects of these parameters on SFE mass extractability [19,21,43].

The observed ERG concentration was higher than the predicted value, lying just above the upper limit of the 95% prediction interval. This is consistent with the high sensitivity of ERG solubility to very small changes in co-solvent at low ethanol levels and, because experimentally no EtOH was used and the model suggested the use of a very low but impactful co-solvent flow, the prediction of ERG concentration was underestimated.

The ERG yield was calculated from the model-predicted mass extraction yield and ERG concentration (product of responses), rather than modeled separately. The experimental ERG yields closely matched the calculated values, indicating that the model captured total ERG recovery well.

Morales et al. reported a higher mass extraction yield than the values obtained in this work, indicating 3.28 g_extract_/100 g_DM_ at 350 bar and 70 °C for 3 h and without co-solvent [21]. However, the ERG concentration in their extract was lower, 180 mg_ERG_/g_extract_. In a later study, the same author applied a sequential extraction method and observed that the lowest mass extraction yield corresponded to the SFE fraction extracted at 350 bar and 40 °C for 3 h, with only 1.1 g_extract_/100 g_DM_ [22]. Despite this low mass yield, this fraction contained the highest ERG concentration, at 88.7 mg ERG/g extract. In another study, Morales et al. performed a large-scale SFE at 225 bar and 40 °C for 3 h. In this case, a more concentrated extract was obtained, with 530 mg_ERG_/g_extract_ [23]. However, the authors highlighted that the scale of the extraction plant influenced the selectivity toward sterols, and that could explain the difference between our work when using a lab-scale extractor.

With our study, it was possible to obtain highly concentrated ERG extracts using SFE without co-solvent, supported by a model with good predictive capacity for the selected responses. The results demonstrate that high pressure enhances ERG concentration in the extracts, while temperature plays a secondary role by improving mass extraction yield, thereby facilitating extract recovery.

#### 2.2.2. Supercritical Fluid Extraction vs. Soxhlet Extraction for Shiitake and Oyster Mushroom

**Figure 2:**
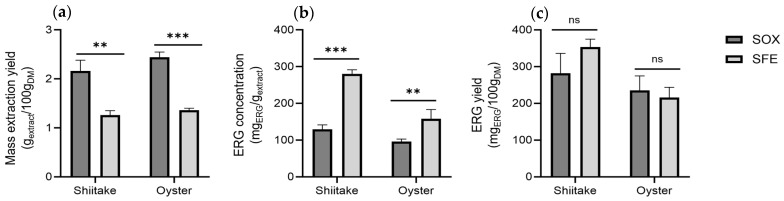
Comparison of the mass extraction, yields (**a**), ERG concentration in the extracts (**b**) and overall ERG yield (**c**) when using SFE and SOX for the shiitake and oyster mushroom (*n* = 3). Significance levels are indicated as follows: ** *p* < 0.01, *** *p* < 0.001; ns = not significant. All experiments were performed in triplicate (*n* = 3).

After reaching the optimal conditions for maximizing the ERG concentration in the extract, the same conditions were applied to the oyster mushroom, and a comparison between the SFE and SOX extraction was assessed. Figure 2 summarizes the results obtained.

Regarding mass extraction yield (Figure 2a), SOX extraction produced higher extract mass than SFE. For shiitake, SOX produced 2.16 ± 0.22 _gextract_/100 g_DM_ and SFE produced 1.26 ± 0.09 g_extract_/100 g_DM_. SOX produced 2.44 ± 0.11 _gextract_/100 g_DM_ and SFE produced 1.36 ± 0.04 g_extract_/100 g_DM_ for oyster mushroom. This was expected, as *n*-hexane can solubilize a broader range of lipophilic compounds than CO_2_, resulting in a less selective extraction and, consequently, higher total extract mass. Although the difference was small, the mass extraction yield was slightly higher for oyster mushroom than for shiitake, regardless of the extraction technique. This observation may be related to differences in cell wall composition. Shiitake mushrooms are reported to contain higher levels of chitin, resulting in a more rigid cell wall structure, which could hinder solvent penetration and reduce mass extractability compared to oyster mushrooms [44,45,46,47].

Regarding ERG concentration in the extracts (Figure 2b), SFE produced significantly more concentrated extracts than SOX extraction for both mushroom species. Notably, the extract concentrations increased by 107% for the shiitake mushroom (129.56 ± 11.89 mg_ERG_/g_extract_ with SOX and 280.57 ± 10.80 mg_ERG_/g_extract_ with SFE) and 65% in oyster mushroom (95.87 ± 7.18 mg_ERG_/g_extract_ with SOX and 158.21 ± 24.85 mg_ERG_/g_extract_ with SFE), when using SFE compared to SOX. This can be attributed to the higher selectivity of supercritical CO_2_, particularly in the absence of a co-solvent, which solubilizes non-polar compounds such as ERG, while limiting the co-extraction of other matrix components. A species-dependent effect was also observed, with shiitake producing more ERG-rich extracts than oyster mushroom. This is consistent with its higher intrinsic ERG content, which appears to outweigh potential limitations associated with its more rigid cell wall structure. It should also be noted that the SFE conditions were optimized specifically for shiitake and therefore may not represent optimal conditions for oyster mushroom.

ERG yields (Figure 2c) of 282.31 ± 54.00 mg_ERG_/100 g_DM_ and 235.04 ± 28.06 mg_ERG_/100 g_DM_ were recovered by SOX for shiitake and oyster mushroom, respectively, while SFE yielded 353.53 ± 21.36 mg_ERG_/100 g_DM_ and 215.69 ± 39.89 mg_ERG_/100 g_DM_. No significant differences were observed between techniques for either species, indicating comparable total ERG recovery. This equivalence arises from opposing behaviors: SOX produces higher total extract mass at lower ERG concentration, while SFE yields more concentrated extracts at lower overall mass. To the best of our knowledge, published data on SFE for oyster mushroom ERG extraction remains scarce. However, the extracts obtained here were considerably more concentrated than those reported by Milovanovic et al. [48] for a mushroom of the same genus, demonstrating SFE’s potential for this species.

This finding highlights the advantage of high-pressure SFE as a more selective and environmentally friendly technique, capable of achieving similar ERG recovery while producing extracts of higher purity. However, the impact of the high pressures on energy costs needs to be further evaluated.

Given the potential limitations imposed by the rigid fungal cell wall, the application of sample pre-treatments to enhance ERG extraction was explored in the following section.

### 2.3. Sample Pre-Treatments to Enhance Ergosterol Extraction

The influence of sample pre-treatments—ultrasound (US), microwave (MW), and enzymatic (EZ)—on the ERG concentration in the extracts was evaluated. Shiitake mushroom samples were subjected to the respective pre-treatments and subsequently extracted using conventional methods. The results obtained from pre-treated samples were compared with untreated controls.

Based on these results, the most effective pre-treatments were selected and applied in combination with SFE for shiitake mushroom. The pre-treatment that provided the best performance under SFE conditions for shiitake was then also applied to the SFE of oyster mushroom.

#### 2.3.1. Sample Pre-Treatments and Conventional Extraction

For the US and MW, after the pre-treatment, the treated biomass was submitted to SOX and compared to SOX without pre-treatment. In the case of EZ, after the pre-treatment, a solid–liquid (SL) extraction was performed instead of SOX and compared to a SL without pre-treatment. The EZ pre-treatment was performed on a small scale (due to availability and cost associated with chitinase) and due to technical limitations, SOX was undoable. Nevertheless, the solvent, extraction time, and solid-to-solvent ratio were kept identical to those used for SOX. This methodological difference should be considered when interpreting the performance of the EZ pre-treatment relative to US and MW. Since EZ-treated samples were benchmarked against untreated SL control rather than SOX, direct quantitative comparisons across pre-treatment strategies should be interpreted with caution. Statistical analysis indicates that the two baselines are reasonably aligned in relative terms, although not strictly equivalent (see Supplementary Information, Table S3). Nonetheless, these findings do not compromise the validity of the reported results within each experimental framework, as all comparisons were performed against their respective untreated controls under consistent conditions.

**Figure 3:**
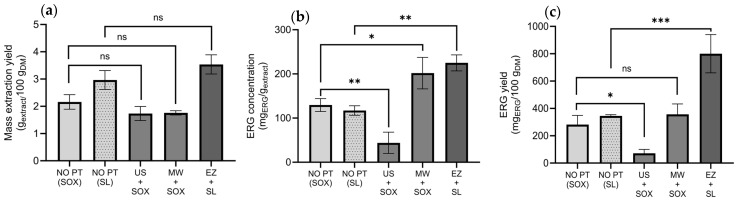
Comparison of mass extraction yield (**a**), ERG concentration in the extracts (**b**), and ERG yield (**c**) for US, MW, and EZ pre-treatments relative to their respective untreated controls (NO PT). US and MW pre-treated samples were compared with SOX without pre-treatment, while EZ pre-treated samples were compared with SL without pre-treatment. Statistical comparisons were performed using unpaired two-tailed *t*-tests between each pre-treated sample and its respective control. Significance levels are indicated as follows: * *p* < 0.05, ** *p* < 0.01, *** *p* < 0.001; ns = not significant. All experiments were performed in triplicate (*n* = 3).

Results for the mass extraction yield, the ERG concentration in the extracts and the ERG yield for each pre-treatment and their respective untreated controls (NO PT) are summed up in Figure 3.

Regarding mass extraction yield (Figure 3a), 1.74 ± 0.21 g_extract_/100 g_DM_ for US, 1.76 ± 0.06 g_extract_/100 g_DM_ for MW and 3.54 ± 0.29 g_extract_/100 g_DM_, no statistically significant differences were observed between pre-treated samples and their respective untreated controls (SOX—2.16 ± 0.22 g_extract_/100 g_DM_ and SL—2.97 ± 0.25 g_extract_/100 g_DM_), indicating that none of the applied pre-treatments significantly influenced overall extractability under the conditions studied.

Regarding ERG concentration (Figure 3b), the three pre-treatments produced distinct outcomes. US pre-treatment (53.36 ± 17.75 mg_ERG_/g_extract_) resulted in a significant decrease relative to the untreated (129.56 ± 11.89 mg_ERG_/g_extract_), suggesting that the applied conditions negatively affected ERG extraction. This may be attributed to the lower cavitation intensity generated by the ultrasonic bath, which delivers less energy to the matrix and results in insufficient cell wall disruption. Furthermore, the shear forces and free radicals produced during acoustic cavitation may have contributed to the degradation of ERG, reducing its concentration in the final extract [49,50].

In contrast, MW pre-treatment significantly increased ERG concentration (201.79 ± 29.05 mg_ERG_/g_extract_), 56% more than the untreated SOX. This suggests that partial disruption of the fungal cell wall improved ERG accessibility while limiting the co-extraction of non-target compounds [31].

Within the extraction conditions evaluated for EZ, enzymatic pre-treatment produced the largest increase in ERG concentration relative to its untreated SL control (225.06 ± 14.85 mg_ERG_/g_extract_ vs. 117.17 ± 7.58 mg_ERG_/g_extract_), 92% more than the untreated SL, consistent with chitinase-mediated disruption of the chitin-rich cell wall and enhanced release of intracellular ERG [51,52]. Chitinase-mediated hydrolysis of chitin generates water-soluble degradation products, including chitooligosaccharides and N-acetylglucosamine monomers, which may accumulate in the aqueous phase surrounding the biomass [52]. The sequential washing and centrifugation steps applied after EZ, mainly to exclude the enzyme, likely contributed to the removal of these water-soluble compounds alongside other polar matrix components [35], potentially improving the relative enrichment of ERG in the pellet prior to subsequent *n*-hexane extraction. However, based on the present data, the specific contribution of this selective depletion to the overall improvement in ERG concentration cannot be fully isolated from the effect of enzymatic cell wall disruption.

Regarding ERG yield (Figure 3c), US pre-treatment (72.28 ± 22.99 mg_ERG_/g_extract_) resulted in a significant decrease compared with the untreated SOX (282.31 ± 54.00 mg_ERG_/g_extract_), consistent with its negative effect on ERG concentration. MW pre-treatment (356.87 ± 62.09 mg_ERG_/g_extract_) did not produce a statistically significant change in ERG yield, due to the balance achieved with less mass extraction yield and higher ERG concentration in the extracts. EZ pre-treatment (800.22 ± 114.20 mg_ERG_/100 g_DM_) resulted in a significant increase of 131% in ERG yield compared with the untreated SL (345.54 ± 6.25 mg_ERG_/100 g_DM_), reflecting the simultaneous increases observed in both mass extraction yield and ERG concentration.

Based on these results, MW and EZ pre-treatments were selected for further combination with SFE. Despite the absence of a significant effect on ERG yield, MW was retained due to its significant enhancement of ERG concentration, which is the primary objective of this work.

#### 2.3.2. Sample Pre-Treatments Synergy with Supercritical Fluid Extraction

**Figure 4:**
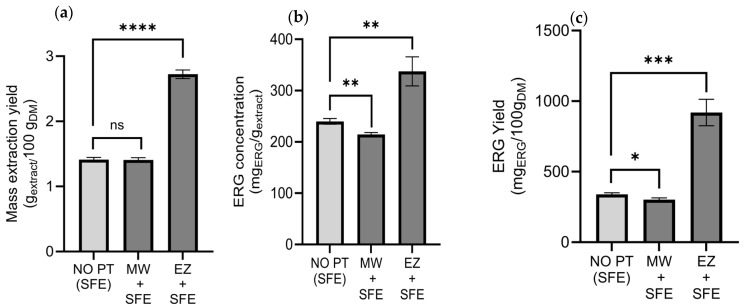
Comparison of mass extraction yield (**a**), ERG concentration in the extracts (**b**), and ERG yield (**c**) obtained by SFE of shiitake mushroom, with and without pre-treatments. MW and EZ pre-treated samples were compared with SFE performed without pre-treatment (NO PT). Statistical comparisons were performed using unpaired two-tailed *t*-tests between each pre-treated sample and the untreated SFE control. Significance levels are indicated as follows: * *p* < 0.05, ** *p* < 0.01, *** *p* < 0.001, **** *p* < 0.0001; ns = not significant. All experiments were performed in triplicate (*n* = 3).

MW and EZ pre-treatments were applied to shiitake mushrooms, followed by SFE under the optimized conditions. Figure 4 summarizes the results obtained.

MW pre-treatment (1.41 ± 0.03 g_extract_/100 g_DM_) did not significantly impact mass extraction yield relative to SFE without pre-treatment (1.41 ± 0.03 g_extract_/100 g_DM_), as it was observed before when comparing to SOX. In contrast, EZ pre-treatment resulted in a highly significant increase in mass extraction yield (2.72 ± 0.05 g_extract_/100 g_DM_), consistent with chitinase-mediated modification of the chitin-rich cell wall, which increases matrix porosity and facilitates supercritical CO_2_ penetration and solute accessibility (Figure 4a).

Regarding ERG concentration (Figure 4b), MW pre-treatment (214.36 ± 3.21 mg_ERG_/g_extract_) resulted in a significant decrease relative to the untreated control (239.96 ± 4.55 mg_ERG_/g_extract_). This contrasts with the positive effect of MW pre-treatment observed when combined with SOX extraction, and the difference between these outcomes is informative. When combined with SOX, MW pre-treatment improved ERG concentration relative to a baseline that is inherently limited by the ability of *n*-hexane to penetrate the intact rigid cell wall through repeated passive contact cycles. In that context, even partial cell wall disruption by MW was sufficient to meaningfully improve ERG accessibility beyond what SOX alone could achieve. Under SFE conditions, however, this effect was not maintained. This may indicate that the structural modifications induced by MW were not sufficient to further improve extraction under conditions already optimized for selective ERG recovery.

In contrast, EZ pre-treatment resulted in a significant increase of 40% in ERG concentration (337.53 ± 23.12 mg_ERG_/g_extract_), suggesting that chitinase-mediated cell wall modification enhances extraction performance when combined with SFE. The enzymatic modification of the fungal cell wall likely increases matrix porosity, enabling supercritical CO_2_ to more effectively access and solubilize intracellular ERG. Furthermore, the washing steps applied after enzymatic treatment may contribute to the removal of polar compounds, potentially improving extract enrichment during subsequent SFE.

Regarding ERG yield (Figure 4c), MW pre-treatment (301.46 ± 10.15 mg_ERG_/100 g_DM_) resulted in a significant decrease compared to SFE without pre-treatment (338.80 ± 9.54 mg_ERG_/100 g_DM_), reflecting its negative effect on ERG concentration without improving mass extraction yield. In contrast, EZ pre-treatment led to a highly significant increase of 171% in ERG yield (919.34 ± 76.1 mg_ERG_/100 g_DM_), consistent with the simultaneous enhancement of both mass extraction yield and ERG concentration. These results indicate that EZ pre-treatment effectively improves overall ERG recovery under SFE conditions.

The use of EZ pre-treatment and SFE created a synergistic effect that further enhanced mass extraction yield, ERG concentration in the extracts and ERG yield. Based on these results, EZ was applied to the oyster mushroom and extracted with SFE.

**Figure 5:**
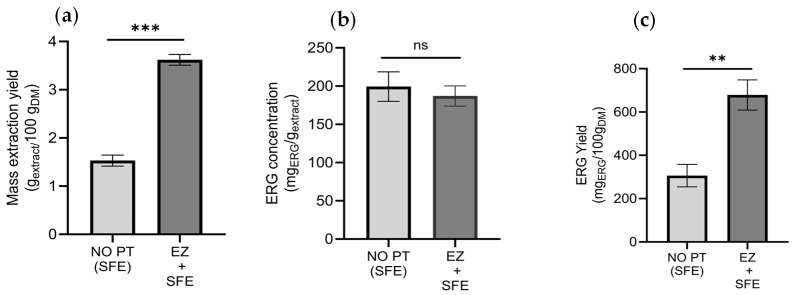
Comparison of mass extraction yield (**a**), ERG concentration in the extracts (**b**), and ERG yield (**c**) obtained by SFE of oyster mushroom, with and without EZ pre-treatments. Statistical comparisons were performed using unpaired two-tailed *t*-tests between each pre-treated sample and the untreated SFE control. Significance levels are indicated as follows: ** *p* < 0.01, *** *p* < 0.001; ns = not significant. All experiments were performed in triplicate (*n* = 3).

Regarding mass extraction yield (Figure 5a), EZ pre-treatment (3.62 ± 0.08 g_extract_/100 g_DM_) produced a highly significant increase relative to SFE without pre-treatment (1.53 ± 0.09 g_extract_/100 g_DM_), consistent with the results observed for shiitake. This observation is consistent with the hypothesis that chitinase-mediated modification of the cell wall, combined with the selective removal of water-soluble compounds during washing, effectively increases the extractable lipophilic material available for supercritical CO_2_ recovery, regardless of mushroom species.

However, EZ pre-treatment did not produce a significant improvement in ERG concentration (Figure 5b) for the oyster mushroom (187.14 ± 9.36 mg_ERG_/g_extract_ for EX + SFE vs. 199.39 ± 15.80 mg_ERG_/g_extract_ for SFE NO PT), in contrast to the significant increase observed for shiitake. This divergent response is likely attributable to the substantially lower chitin content of oyster mushroom compared to shiitake, as consistently reported in the literature [45,47]. In shiitake, chitin represents the dominant structural barrier of the cell wall, and its enzymatic degradation by chitinase substantially improves ERG accessibility. In oyster mushroom, however, the cell wall contains considerably less chitin and is relatively richer in β-glucans [53], which are not substrates for chitinase. The enzymatic pre-treatment therefore targets only a minor structural component of the oyster cell wall, limiting its effectiveness in selectively improving ERG accessibility. Under the optimized SFE conditions applied here, supercritical CO_2_ appears already capable of efficiently accessing intracellular ERG in oyster mushroom without enzymatic assistance, leaving little room for further enrichment. The increase in mass extraction yield without a corresponding gain in ERG concentration further suggests that pre-treatment enhanced the non-selective release of extractable material rather than specifically improving ERG accessibility in this species.

Regarding ERG yield (Figure 5c), EZ pre-treatment resulted in a significant increase of 121% for oyster mushroom, driven primarily by the substantial gain in mass extraction yield rather than by improved ERG concentration (678.69 ± 49.1 mg_ERG_/g_extract_ for EX + SFE vs. 306.11 ± 42.051 mg_ERG_/g_extract_ for SFE NO PT). This indicates that for oyster mushroom, EZ pre-treatment enhances total ERG recovery through increased extractability rather than through selective enrichment, a fundamentally different mechanism from that observed in shiitake.

Taken together, the results across both species demonstrate that the benefit of EZ pre-treatment is matrix-dependent and governed primarily by cell wall chitin content. For chitin-rich matrices such as shiitake, enzymatic pre-treatment acts synergistically with SFE by selectively improving both ERG accessibility and extract enrichment. For matrices with lower chitin content such as oyster mushroom, the optimized SFE conditions alone are sufficient to achieve effective ERG extraction, and EZ pre-treatment offers limited advantage in terms of concentration enrichment, though it does improve total ERG recovery.

## 3. Materials and Methods

### 3.1. Mushroom Samples

Two species of edible mushrooms—*Lentinula edodes* (shiitake mushroom) and *Pleurotus ostreatus* (oyster mushroom)—were provided by Centro de Investigação de Montanha (CIMO), Instituto Politécnico de Bragança (IPB). The samples were already dried and coarsely ground. To ensure particle size uniformity, the samples were further milled with a ball mill (Powteq, 13 BM6pro, Beijing,, China) and refined using a vibratory sieve shaker (Retsch, AS200 basic, Haan, Germany), establishing a target particle size range between 63 µm and 250 µm for the subsequent experiments.

### 3.2. Chemicals

Solvent used for conventional extractions and chromatographic analysis was *n*-hexane for analysis (99%, Carlo Erba, Milano, Italy) and ethanol (98.8%, Honeywell Riedel-de Haën, Seelze, Germany). Solvents used for SFE were CO_2_ pure grade (99.95%, Air Liquide, Lisbon, Portugal) and ethanol (98.8%, Honeywell Riedel-de Haën, Germany). For drying, nitrogen gas (99.95%, Air Liquide, Lisbon, Portugal) was used. For GC-MS analysis, the carrier gas used was helium (99.999%, Air Liquide, Lisbon, Portugal) and the standards used were ergosterol for R&D (96%, Thermo Scientific, Waltham, MA, USA) and cholecalciferol (98%, TCI, Tokyo, Japan). For enzymatic pre-treatment, chitinase from *Streptomyces griseus* (SAE0158-10UN, Sigma-Aldrich, Darmstadt, Germany). Sodium acetate buffer 50 mM pH 5.8 [54] was chosen and prepared using Type II purified water (>5 MΩ·cm), obtained by reverse osmosis using an Elix^®^ Essential Water Purification System (Merck, Darmstadt, Germany), sodium acetate anhydrous (Sigma-Aldrich, St. Louis, MO, USA) and glacial acetic acid (Panreac, Barcelona, Spain).

### 3.3. Conventional Extraction—Soxhlet

For the conventional extraction, 6 g of mushroom powder from each species was subjected to Soxhlet (SOX) extraction, in triplicate. Extraction was conducted using 200 mL of *n*-hexane as the solvent (30 g/L) for 3 h. Following the extraction, the solvent was removed under reduced pressure (Büchi, Rotavapor R-114, Flawil, Switzerland) to obtain dried extracts, which were stored in the dark at −20 °C until further analysis. The mass extraction yield was calculated as grams of extract per 100 g of dry mushroom (g_extract_/100 g_DM_).

### 3.4. Supercritical Fluid Extraction with Carbon Dioxide

Supercritical Fluid Extraction (SFE) was employed to obtain ergosterol (ERG) rich extracts from mushroom biomass using a high-pressure SFE system (SFE Process Extraction, Model SFE Lab, Tomblaine, France). The extraction was carried out in a 100 mL vessel, capable of reaching pressures up to 1000 bar. Liquid CO_2_ was delivered through a high-performance pump, chilled, and subsequently pressurized with a back pressure regulator. The CO_2_ flow rate was maintained at 20 g/min, and the total extraction time was 3 h. The co-solvent was pumped using a high-performance pump. After the extraction, EtOH was passed through the extractor to recover any extract remaining inside. The collected material was then dried under reduced pressure, and the resulting extracts were stored in the dark at −20 °C until further analysis. The mass extraction yield was calculated as grams of extract per 100 g of dry mushroom (g_extract_/100 g_DM_).

#### Experimental Design Analysis for the Supercritical Fluid Extractions

Response Surface Methodology (RSM) was used to optimize SFE. This statistical tool was used to model the effect of temperature, pressure and the use of co-solvent (EtOH) on the mass extraction yield and ERG concentration. A Design of Experiments (DoE) for the extraction was carried out following a Central Composition Face (CCF) design composed of fractional or full factorial design and star points placed on the faces of the sides, as a function of pressure (350–800 bar), temperature (40–70 °C) and co-solvent (0–10 mL/min). After the selection of independent variables and their ranges, experiments were established based on a CCF design, and each one was coded at three levels, i.e., −1 (the lowest of each variable), 0 (middle level), and +1 (the highest of each variable). The DoE and RSM were performed using the software MODDE, version 13.1 32-bit (Umetrics, Malmö, Sweden). A total of 17 experimental runs were performed in a randomized order to minimize the influence of systematic errors, and a center point was included in triplicate to enable the estimation of pure experimental error, independent of model fitting.

### 3.5. Sample Pre-Treatments to Enhance Ergosterol Extraction

#### 3.5.1. Ultrasound Pre-Treatment

Ultrasound (US) pre-treatment was performed using an ultrasonic bath system (Argo LAB, DU-32, Carpi, Italy) and applied to 6 g of shiitake mushroom, the mass necessary for subsequent SOX extraction. A volume of 40 mL of EtOH (the necessary amount to wet the biomass) was used and the pre-treatment duration was 15 min, based on the conditions previously reported by Heleno et al. [15]. The temperature was set to 70 °C to match the SOX conditions temperature to avoid additional thermal stress that could degrade ERG. The US power was set to 120 W, the highest permitted by the device, to ensure adequate energy distribution and to allow the target temperature to be reached within a reasonable timeframe. The treatment was conducted in triplicate. Following the pre-treatment, the biomass was dried with nitrogen, and the pre-treated biomass was stored in the dark at −20 °C until further analysis.

#### 3.5.2. Microwave Pre-Treatment

Microwave (MW), Discover SP-D Microwave System, (CEM, Mattews, NC, USA) pre-treatment was applied to 6 g of shiitake mushroom, the mass necessary for subsequent SOX extraction. A volume of 40 mL of EtOH (the necessary amount to wet the biomass) was selected and the pre-treatment duration was 15 min, closely matching Heleno et al. [7] previously reported conditions and US pre-treatment. The temperature was set to 70 °C to match SOX conditions, and to avoid additional thermal stress that could degrade ERG. MW maximum power was set to 300 W [7]. The treatment was conducted in triplicate. Following the pre-treatment, the samples were dried with nitrogen, and the pre-treated biomass was stored in the dark at −20 °C until further analysis.

#### 3.5.3. Enzymatic Pre-Treatment

Enzymatic pre-treatment was performed on 0.5 g of mushroom biomass, using chitinase from *Streptomyces griseus* (SAE0158-10UN, Sigma-Aldrich, Germany). Enzymatic activity for the batch (0000104386) used was 634 U/g_solid_, where one unit (U) liberates 1 mg of N-acetyl-D-glucosamine from chitin per hour.

An enzyme loading of 0.25 U/g_DM_ was selected based on preliminary experiments in which a range of concentrations (0.25–1.5 U/g_DM_) was explored. As the aim of this pre-treatment was to partially disrupt the fungal cell wall rather than achieve complete hydrolysis, low enzyme loadings were deliberately investigated. A loading of 0.25 U/g_DM_ biomass was selected as it provided the best ERG recovery while minimizing enzyme consumption. Each treatment was carried out in 10 mL of 50 mM sodium acetate buffer (pH 5.8), widely reported for chitinase activity as typical exhibits optimal performance under mildly acidic conditions close to this pH [54,55,56]. Incubation was performed in a water bath at 37 °C for 2 h, optimal conditions expressed on the product data information of the supplier, under magnetic stirring (200 rpm).

Following incubation, samples were mixed with 20 mL of cold distilled water and centrifuged at 4 °C, 10,000 rpm for 10 min. The pellet was washed three times with cold distilled water and centrifuged after each wash. Supernatants were discarded while the wet pellet was dried under reduced pressure using a vacuum concentrator (Centrivap, Labconco, Kansas, MO, USA). The pre-treated biomass was subjected to a solid–liquid extraction with *n*-hexane under the same conditions as SOX extraction applied to the other pre-treatments. The mass-to-solvent ratio was maintained at 30 g/L, extraction was performed at ~70 °C for 3 h, and biomass was separated from the *n*-hexane phase by vacuum filtration using a qualitative filter. The *n*-hexane fraction was evaporated at reduced pressure, and the resulting extracts were stored at −20 °C in the dark until further analysis.

### 3.6. Ergosterol Quantification

All the extracts were analyzed to quantify ERG by GC-MS. Direct GC-MS analysis of non-derivatized ergosterol was performed, as reliable chromatographic separation and identification were achieved under the selected analytical conditions, consistent with a previous report [57]. The extracts were dissolved in *n*-hexane or ethanol, with concentrations ranging from 0.20 to 1 mg_extract_/mL, and cholecalciferol (0.06 mg/mL) was added as an internal standard. GC-MS analysis was performed using a GCMS-QP2010 Plus chromatograph (Shimadzu, Kyoto, Japan) coupled with an autosampler AOC-5000 (Shimadzu). A 5-MS capillary column (30 m length × 0.25 mm i.d. × 0.25 μm film thickness, Teknokroma, Barcelona, Spain) was used to separate the compounds using He (≥99.999% purity) as the carrier gas at a flow rate of 2 mL/min. GC program was set as follows: column temperature was initially maintained at 120 °C for 3 min, then increased at a rate of 20 °C per minute to 290 °C and held for 5 min, and, finally, to 320 °C at a rate of 30 °C per minute (held for 5 min). Splitless injection of 1 µL of sample was done at 290 °C (injector temperature). The ion source and interface temperatures were kept at 280 °C. Mass spectra were recorded in electron ionization (EI) mode at 70 eV with a scan range of 40–400 *m*/*z* and a speed of 769 scans/s and selection monitoring (SIM) at 363 *m*/*z* (quantification) and 337 *m*/*z* (qualification) for ERG and 351 *m*/*z* (quantification) and 325 *m*/*z* (qualification) for cholecalciferol (internal standard).

Quantification was made through two different calibration curves of ERG from 0.005 to 0.180 mg/mL using two different types of solvents: ethanol for quantifying the SFE extracts and *n*-hexane for all the other extracts. For both calibration curves, the internal standard concentration was constant (0.06 mg/mL). Data acquisition and processing were performed using LabSolutions, GCMSsolution software (version 4.50 SP1, Shimadzu Corporation, Kyoto, Japan). The ERG content of the extracts analyzed was expressed in milligrams of ERG per gram of extract (mg_ERG_/g_extract_).

Calibration curves for ERG quantification in both n-hexane and ethanol, including the linear regression equations, coefficients of determination (r^2^), and representative chromatograms, are presented in the Supplementary Information (Figures S2–S4).

### 3.7. Statistical Analysis

Statistical analysis regarding the Design of Experiments was performed using MODDE software.

Statistical analysis was performed using GraphPad Prism software, version 10.4.1 (GraphPad Software, San Diego, CA, USA). Differences between the two extraction techniques and pre-treated samples and their respective untreated controls were evaluated using unpaired two-tailed Student’s *t*-tests. Results are expressed as mean ± standard deviation (SD) of triplicate experiments (*n* = 3), and statistical significance was considered at *p* < 0.05.

## 4. Conclusions

This work demonstrated that supercritical CO_2_ extraction is a more selective and environmentally friendly alternative to conventional Soxhlet (SOX) extraction to produce ERG-rich extracts from shiitake and oyster, achieving significantly higher ERG concentrations while maintaining comparable overall ERG recovery. RSM-based optimization highlighted the value of exploring extended high-pressure ranges beyond those reported in the literature. Among the cell wall pre-treatments evaluated, enzymatic pre-treatment with chitinase was the only strategy to act synergistically with SFE, improving both ERG concentration and yield in shiitake through a combination of chitin-specific cell wall modification. The species-dependent nature of this effect underlines the importance of matching pre-treatment strategies to the cell wall composition of the target matrix. Collectively, these findings establish high-pressure SFE combined with chitinase pre-treatment as an effective and selective combination for obtaining ERG-rich extracts from chitin-rich fungal matrices. These findings represent a step forward towards the implementation of green techniques that could be applied to misshapen and unmarketable mushrooms and contribute to valorizing underutilized biomass. Despite the promising results obtained, some limitations regarding industrial implementation should be considered. The use of very high operating pressures may increase capital and energy costs, since many industrial SFE systems commonly operate at lower pressure ranges. Therefore, the improvement in extract selectivity achieved at high pressure should be balanced against process economics. Likewise, the enzymatic pre-treatment may present additional challenges related to enzyme cost, process integration, and scale-up logistics, although strategies such as enzyme recycling or continuous processing may improve feasibility. Future work should therefore address techno-economic aspects and scalability of both the high-pressure SFE process and enzymatic pre-treatment protocol.

## Figures and Tables

**Table 1:** Mass extraction yield, ERG concentration and ERG yield of the extracts obtained by SOX extraction for the two types of mushrooms. Results are expressed as mean ± SD, *n* = 3. DM—dry mushroom.

Mushroom	Mass Extraction Yield (g_extract_/100 g_DM_)	ERG Concentration (mg_ERG_/g_extract_)	ERG Yield (mg_ERG_/100 g_DM_)
Shiitake	2.16 ± 0.22	129.56 ± 11.89	282.31 ± 54.00
Oyster	2.44 ± 0.11	95.87 ± 7.18	235.04 ± 28.06

**Table 2:** Design of Experiments varying pressure, temperature and co-solvent. Mass extraction yield and ERG concentration were the responses evaluated.

	Variables			Responses	
No.	Pressure (bar)	Temperature (°C)	Co-Solvent (mL/min)	Mass Extraction Yield (g_extract_/100 g_DM_)	ERG Concentration (mg_ERG_/g_extract_)
1	350	40	0	0.9	237.53
2	350	40	10	5.04	163.74
3	350	55	5	2.27	117.63
4	350	70	0	1.06	234.86
5	350	70	10	6.41	130.51
6	575	40	5	2.82	176.76
7	575	55	0	0.85	224.44
8	575	55	5	3.96	105.44
9	575	55	5	2.96	163.14
10	575	55	5	4.3	105.81
11	575	55	10	6.48	103.3
12	575	70	5	5.05	125.21
13	800	40	0	0.78	243.57
14	800	40	10	5.49	83.92
15	800	55	5	4.9	121.39
16	800	70	0	1.22	207.12
17	800	70	10	5.74	105.05

**Table 3:** Predicted values of the model for the mass extraction yield and the ERG concentration and the corresponding observed value. The results are presented as mean ± SD, *n* = 3.

	Mass Extraction Yield (g_extract_/100 g_DM_)	ERG Concentration (mg_ERG_/g_extract_)	ERG Yield (mg_ERG_/100 g_DM_)
Predicted	1.56	221.27	345.18
Observed	1.29 ± 0.09	280.57 ± 10.80	353.53 ± 21.36

## Data Availability

The original contributions presented in this study are included in the article/Supplementary Materials. Further inquiries can be directed to the corresponding author.

## Supplementary Materials

The following supporting information can be downloaded at: https://www.mdpi.com/article/10.3390/molecules31122067/s1; Table S1: factors significance of the Design of Experiments, Table S2: model statistical analysis of RSM, Table S3: comparision of the results of SOX and SL, Figure S1: representative GC-MS chromatograms of the mixtures of ERG standard and internal standard, Figure S2: calibration curves of mixture (ERG standard and internal standard), Figure S3: GC-MS chromatogram of an SFE extract (TIC), and Figure S4: GC-MS chromatogram of an SFE extract (SIM).

## Abbreviations

ERG	Ergosterol
SFE	Supercritical Fluid Extraction
EtOH	Ethanol
US	Ultrasound
MW	Microwave
EZ	Enzymatic
SOX	Soxhlet
DM	Dry Mushroom
DoE	Design of Experiments
RSM	Response Surface Methodology
SL	Solid–Liquid
NO PT	No Pre-Treatment
