# Supplementary material for: Supercritical Fluid Extraction of Ergosterol from Lentinula edodes and Pleurotus ostreatus: Optimization and Synergistic Effects of Biomass Pre-Treatments
Source: Molecules. 2026 Jun 12;31(12):2067. doi: 10.3390/molecules31122067 (PMC13306169; doi:10.3390/molecules31122067)
Supplement: Supplementary file 1 [file molecules-31-02067-s001.zip › molecules-4332207-supplementary.pdf]

# Supercritical Fluid Extraction of Ergosterol from *Lentinula edodes* and *Pleurotus ostreatus*: Optimization and Synergistic Effects of Biomass Pre-treatments

**Rita Faustino [1], António Ferreira [1], Maria Rosário Bronze [1,2,3] and Naiara Fernández [1*]**

[1] Instituto de Biologia Experimental e Tecnológica, Apartado 12, 2881-901 Oeiras, Portugal;
[2] Instituto de Tecnologia Química e Biológica António Xavier, Universidade Nova de Lisboa, Av. República, 2780-157 Oeiras, Portugal;
[3] FFULisboa, Faculdade de Farmácia da Universidade de Lisboa, Av. Professor Gama Pinto, 1649-003 Lisboa, Portugal;
* Correspondence: naiara.fernandez@ibet.pt

## Supplementary Information

### 1. Experimental design and statistical analysis

A response surface methodology was used to evaluate the influence of process conditions on the mass extraction yield and ergosterol concentration in the extracts, obtained using Supercritical Fluid Extraction (SFE).
The effect of pressure (350 bar – 800 bar), temperature (40°C – 70°C) and co-solvent flow (ethanol, 0 ml/min – 10 mL/min) on mass extraction yield and ergosterol (ERG) concentration in the extracts, were studied.

The mass extraction yield and ERG concentration in the extracts data resulting from the experiments performed according to the experimental conditions defined by the Central Composite Face (CCF) design, were analyzed by using the MODDE (Version 13.1 32- bit, Umetrics, Sweden) software. The statistical tests, including the adjustments of the design model and factors effects, were significant when the resulting $p$-value was lower than the predefined $\alpha$ = 0.05.
The underlying three-factor polynomial models include linear, two-factor interactions as well as quadratic terms as depicted by Equation (S1). In this equation, P, T, and C represent independent variables, i.e., pressure, temperature, and co-solvent flow.

$$Y = b_0 + b_1T + b_2P + b_3C + b_{11}T^2 + b_{22}P^2 + b_{33}C^2 + b_{12}(TP) + b_{13}(TC) + b_{23}(PC) \qquad \text{(S1)}$$

The model coefficients $(b_0, b_i, b_{ij})$ were estimated by multivariate linear regression, and their significance, as well as the adequacy of the fitted models, were assessed by analysis of variance (ANOVA).

**Table S1-Factors significance of the Design of Experiments.**

Linear, quadratic and interaction effects and respective significant levels (p-value) of the test variables [factors: Pressure (P), temperature (T) and co-solvent flow (C)] and interaction on mass extraction yield and ERG concentration in the extracts.

| | Mass extraction yield | | ERG concentration | |
|---|---|---|---|---|
| | Coeff. SC | *p*-value | Coeff. SC | *p*-value |
| $b_0$ | 3.54 | 8.45E-12 | 125.16 | 6.81E-08 |
| P | | | -12.32 | 0.10 |
| T | 0.45 | 0.07 | -10.28 | 0.17 |
| C | 2.44 | 3.87E-08 | -56.10 | 9.76E-06 |
| $T^2$ | | | 19.65 | 0.15 |
| $C^2$ | | | 32.53 | 0.03 |
| PC | | | -10.45 | 0.20 |

The response surfaces fitted to the mass extraction yield and ERG concentration (**Manuscript Figure 1**) can be described using a polynomial model as a function of pressure (P), temperature (T) and co-solvent flow (C). Non-significant effects (**Table S1**) were maintained on complete model (Equation (**1**)) to preserve model hierarchy, whereby lower-order terms are maintained when higher-order terms involving the same factors are present, ensuring mathematical coherence of the response surface, and predictive capability, resulting in polynomial models described in Equation (S2) and Equation (S3).This approach also preserves terms with potential physical relevance to the supercritical fluid extraction process, such as pressure, whose borderline effect on ERG concentration is consistent with the known influence of $CO_2$ density on solvating power at elevated pressures.

Mass extraction yield ($g_{extract}$/100 $g_{DM}$) = 3.54 + 0.45T + 2.44C (S2)

ERG concentration ($mg_{ERG}/g_{extract}$) = 125.16 -12.32P-10.28T 56.10C+19.65$T^2$+32.53$C^2$-10.45PC (S3)

The ANOVA results for the fitted model of mass extraction yield and ERG concentration in the extracts are presented in **Table S2.**

**Table S2-Model statistical analysis of RSM.**

ANOVA analysis for mass extraction yield and ERG concentration in the extract, when varying pressure, temperature and co-solvent.

| **Mass extraction Yield** | **DF** | **SS** | **MS (variance)** | **F** | **p** | **SD** |
|---|---|---|---|---|---|---|
| Total | 17 | 281.872 | 16.5807 | | | |
| Constant | 1 | 213.391 | 213.391 | | | |
| Total corrected | 16 | 68.4803 | 4.28002 | | | 2.06882 |
| Regression | 2 | 61.2725 | 30.6362 | 59.5056 | **0.000** | 5.535 |
| Residual | 14 | 7.20785 | 0.514847 | | | 0.717528 |
| Lack of Fit (Model error) | 12 | 6.23745 | 0.519788 | 1.07129 | **0.580** | 0.720963 |
| Pure error (Replicate error) | 2 | 0.9704 | 0.4852 | | | 0.696563 |
| | N = 17 | **Q2 =** | **0.863** | Cond. no. = | 1.304 | |
| | DF = 14 | **R2 =** | **0.895** | RSD = | 0.7175 | |
| | | **R2 adj. =** | **0.880** | | | |
| Ergosterol concentration | DF | SS | MS (variance) | F | p | SD |
| Total | 17 | 461193 | 27129 | | | |
| Constant | 1 | 412908 | 412908 | | | |
| Total corrected | 16 | 48285.3 | 3017.83 | | | 54.9348 |
| Regression | 6 | 43573.8 | 7262.29 | 15.414 | 0.000 | 85.2191 |
| Residual | 10 | 4711.5 | 471.15 | | | 21.706 |
| Lack of Fit (Model error) | 8 | 2506.12 | 313.265 | 0.284091 | 0.920 | 17.6993 |
| Pure error (Replicate error) | 2 | 2205.39 | 1102.69 | | | 33.2068 |
| | N = 17 | **Q2 =** | **0.743** | Cond. no. = | 3.978 | |
| | DF = 10 | **R2 =** | **0.902** | RSD = | 21.71 | |
| | | **R2 adj. =** | **0.844** | | | |

The values for $R^2$ of these models suggest a good agreement between the experimental data and the values predicted by the model for the mass extraction yield and efficiency. About 88% and 94% of the observed overall variance concerning the mass extraction yield and ERG concentration respectively, are explained by these models (**Table S2**). The reproducibility of the models for mass extraction yield and ERG concentration was 89% and 63%, respectively, considering the center points. Although the reproducibility of the ERG concentration model was lower and the corresponding RSD value was higher, this was expected due to the inherent variability associated with natural biomasses. Nevertheless, the model showed good statistical performance, with high $R^2$ and $Q^2$ values, as well as a non-significant lack of fit, supporting its adequacy and predictive capability. Model validity values were 0.86 for mass extraction yield and 0.98 for ERG concentration.

## 2. Gas Chromatography-Mass Spectrometry (GC-MS): Chromatograms and Calibration curves

Calibration curves for GC-MS analysis were prepared using mixtures of cholecalciferol (98%, TCI, Tokyo, Japan), used as the internal standard, and an ERG standard (96%, Thermo Scientific, MA, USA) and using Single Ion Monitoring (SIM) mode for acquiring chromatographic data, at *m/z* 363 (quantifier

ion) and *m/z* 337 (qualifier ion) for ERG and *m/z* 351 (quantifier ion) and *m/z* 325 (qualifier ion) for cholecalciferol (internal standard). The concentration of the internal standard was 0.06 mg/mL and ERG concentrations ranged from 0.005 to 0.180 mg/mL.

**Figure S1** presents representative chromatograms of one of the mixtures prepared for the calibration curves. Peak 1 corresponds to cholecalciferol, used as the internal standard, peak 2 corresponds to lumisterol-3, an impurity associated with the internal standard, and peak 3 corresponds to ERG.

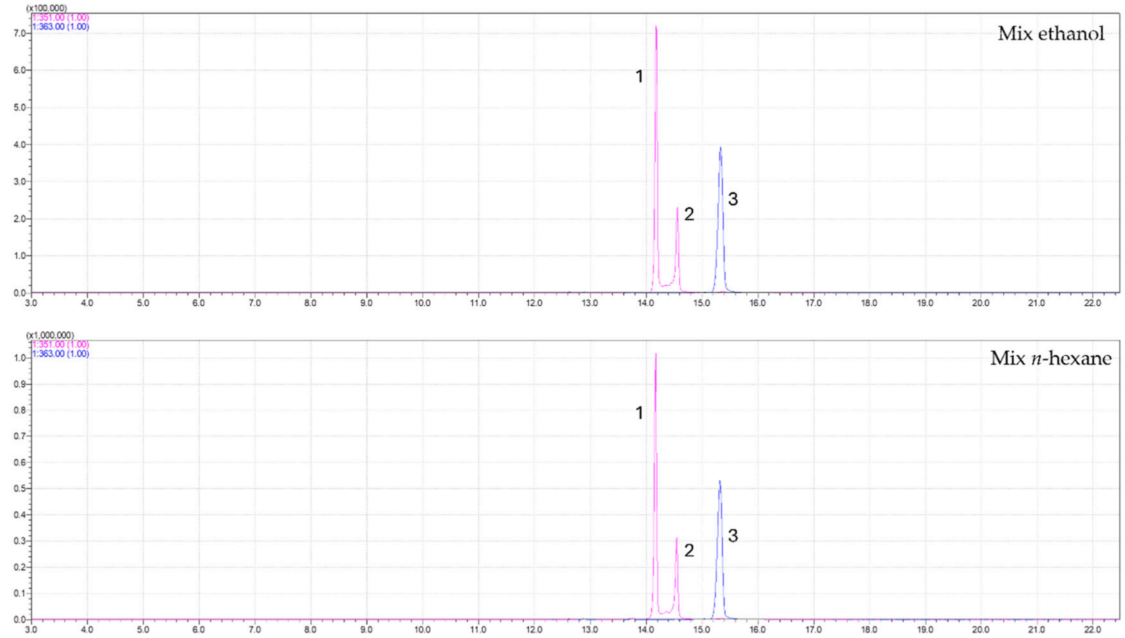


**Figure S1-Representative GC-MS chromatograms of the mixtures of ERG standard and internal standard.** GC-MS SIM chromatogram of the mixtures of the internal standard and the ERG standard, both at 0.06 mg/mL of concentration. SIM acquisition was performed using m/z 351 (quantifier ion) and m/z 325 (qualifier ion) for cholecalciferol (internal standard) and m/z 363 (quantifier ion) and m/z 337 (qualifier ion) for ERG. Peak 1 corresponds to cholecalciferol (RT = 14.23 min), peak 2 corresponds to lumisterol-3 (RT = 14.58 min), and peak 3 corresponds to ERG (RT = 15.36 min).

Calibration curves were prepared using two different solvents, namely ethanol and *n*-hexane. The curves were constructed by plotting the ERG-to-internal standard peak area ratio ($A_{ERG}/A_{IS}$) against ERG concentration (C). **Figure S2** presents the calibration curves obtained for each solvent.

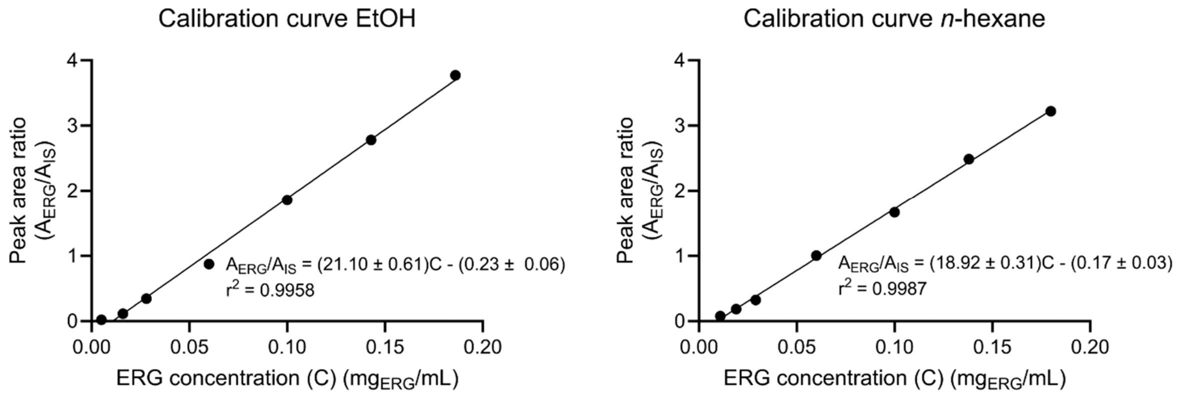


**Figure S2-Calibration curves of Mixture (ERG standard and internal standard).**

Calibration curve for the standard mixture solution containing cholecalciferol and ERG prepared using ethanol (left) and *n*-hexane (right) as solvent. The ERG concentrations varied from [0.005 – 0.180 mg/mL] and cholecalciferol concentration was 0.06 mg/mL. Linear regression was performed for each calibration curve, and the results are expressed as $A_{ERG}/A_{IS}$ = (slope ± u_slope) × C + (intercept ± u_intercept), where $A_{ERG}/A_{IS}$ represents the dependent variable (y) which is the ratio between the ERG peak area and internal standard peak area,, u represents the uncertainty of each regression parameter and C represents the independent variable (x) which is the ERG concentration in $mg_{ERG}$/mL. The coefficient of determination, $r^2$, was obtained through the fitting of the linear regression.

**Figures S3** and **S4** present representative chromatograms of the optimized SFE extract of the shiitake mushroom, obtained with the conditions of 690 bar, 69.8 °C and no co-solvent. The solvent used for analysis was ethanol. **Figure S3** shows the total ion chromatogram (TIC), while **Figure S4** presents the SIM chromatogram used for quantification. In both chromatograms, peak 1 corresponds to cholecalciferol, used as the internal standard, peak 2 corresponds to lumisterol-3, and peak 3 corresponds to ERG, the target compound.

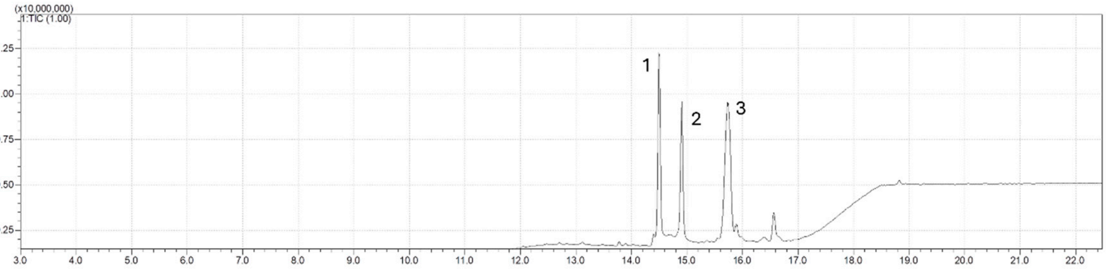


**Figure S3-GC-MS chromatogram of a SFE extract (TIC).**

GC-MS TIC chromatogram of the optimized SFE extract obtained from shiitake mushroom. Peak 1 corresponds to cholecalciferol (internal standard) with a retention time of 14.50 min. Peak 2 corresponds to lumisterol-3, an impurity associated with the internal standard, with a retention time of 14.91 min. Peak 3 corresponds to ERG, with a retention time of 15.74 min.

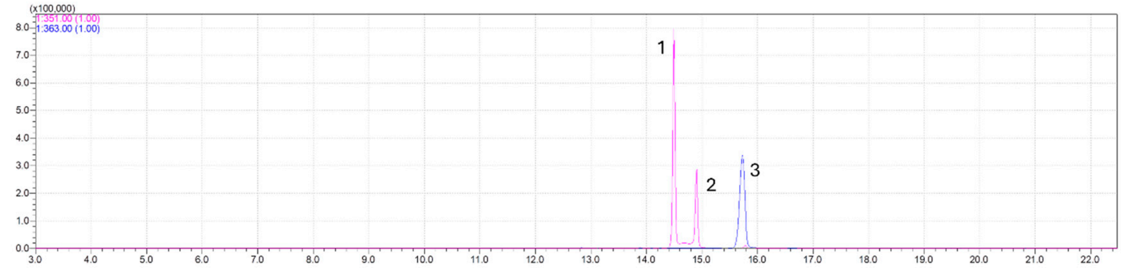


**Figure S4-GC-MS chromatogram of a SFE extract (SIM).**

GC-MS SIM chromatogram of the optimized SFE extract obtained from shiitake mushroom. SIM acquisition was performed using m/z 351 (quantifi*er ion*) and m/z 325 (qualifi*er ion*) for cholecalciferol (internal standard) and m/z 363 (quantifi*er ion) and m*/z 337 (qualifi*er ion*) for ERG. Peak 1 corresponds to cholecalciferol (RT = 14.50 min), peak 2 corresponds to lumisterol-3 (RT = 14.91 min), and peak 3 corresponds to ERG (RT = 15.74 min).

## 3. Sample pre-treatments for enhancing ERG extraction

To avoid overstatement of the EZ pre-treatment performance through cross-comparison, the comparability of the untreated SOX and untreated SL baselines was evaluated. Unpaired t-tests were conducted across the three response variables to assess whether statistically significant differences existed between the two extraction methods.

In addition, to evaluate the magnitude of any differences, a conversion factor was calculated based on the ratio between the mean values of the untreated SOX replicates and those of the untreated SL replicates. **Table S3** summarizes these results.

**Table S3-Comparision of the results of SOX and SL.**

Mean ± standard deviation (SD) (n = 3) for the untreated SOX control (NO PT (SOX)) and untreated SL control (NO PT (SL)). *P*-values obtained from unpaired t-tests performed for each response are reported. The conversion factor between the two techniques was calculated as the ratio of the mean value of the SOX control to that of the SL control.

| Response | NO PT (SOX) Mean ± SD | NO PT (SL) Mean ± SD | *p*-value | Correction factor (SOX/SL) |
|---|---|---|---|---|
| Mass extraction yield | 2.16 ± 0.22 | 2.97 ± 0.25 | 0.121 | 0.73 |
| ERG concentration | 129.56 ± 11.89 | 117.17 ± 7.58 | 0.358 | 1.11 |
| ERG yield | 282.31 ± 54.00 | 345.54 ± 6.25 | 0.238 | 0.82 |

The comparison between the untreated SOX and SL extraction controls demonstrates that no statistically significant differences were observed for any of the evaluated responses, as indicated by p-values greater than 0.05 in all cases. Mass extraction yield, ERG concentration and ERG yield showed comparable values between the two extraction methods under untreated conditions.

Despite the absence of statistical significance, the calculated conversion factors (SOX/SL) suggest some variability in magnitude between the two baselines. Mass extraction yield and ERG yield exhibited factors below unity (0.73 and 0.82, respectively), indicating that SL extraction yielded slightly higher values for these responses. In contrast, ERG concentration showed a factor slightly above unity (1.11), suggesting marginally higher values for SOX extraction. However, they remain within the same order of magnitude (≈1) and do not indicate a systematic bias favoring one extraction method across all responses.

Overall, these results indicate that while the two untreated extraction methods are not strictly identical, they are comparable and do not exhibit a consistent directional bias across all responses. This supports

their use as separate but reasonably aligned baselines, although comparisons across pre-treatment strategies using different controls should be interpreted with caution.
